# Effect of sample volume and time on rumen juice analysis in cattle

**DOI:** 10.1111/jvim.16697

**Published:** 2023-04-07

**Authors:** Suzanne A. C. Clergue, Sarah M. Depenbrock, Munashe Chigerwe

**Affiliations:** ^1^ Livestock Service, Veterinary Medical Teaching Hospital Nantes‐Oniris Veterinary School Nantes France; ^2^ Department of Veterinary Medicine and Epidemiology University of California Davis Davis California USA

**Keywords:** bovine, forestomach indigestion, protozoa motility, rumen fluid, rumen sampling

## Abstract

**Background:**

Rumen juice analysis (RJA) involves analysis of a 10mL sample within minutes after sampling. However, it can be challenging to collect 10 mL of rumen juice (RJ) from some ruminants, and clinical circumstances can delay RJA.

**Objectives:**

Quantify the effect of sample volume (2, 5, 10, 50, and 100 mL), and time‐to‐analysis (0, 30, and 60 minutes) on RJA.

**Animals:**

Cannulated cow.

**Methods:**

Observational experimental study. Two liters of RJ were collected at 26 separate times. The samples were subdivided into 2 duplicates of each sample volume at each sampling time; and analyzed at 0, 30, and 60 minutes after collection. Rumen juice analysis included pH measurement, methylene blue reduction time (MBRT), and protozoal motility.

**Results:**

The pH of 2 and 5 mL samples was significantly (*P* = .01) higher than the pH of 50 and 100 mL samples at all time points. The MBRT was significantly lower (faster bacterial reduction) for 100 mL samples compared to all other samples at 0 minute and to 2, 5, and 50 mL samples at 30 min. The pH and MBRT at 60 minutes were significantly higher than at 0 minute for all volumes (*P* < .05 and *P* < .01, respectively). For large protozoa, small sample volumes (2 and 5 mL) had significantly lower protozoal motility (scores of 5 and 4.5, respectively) compared to 100 mL samples at 60 minutes (score of 4; *P* < .05).

**Conclusions and Clinical Importance:**

Interpretation of RJA could be affected by small sample volumes and delays to analysis. Sample volumes of ≥10 mL analyzed within 30 minutes after collection are recommended.

**Abbreviations**
CO_2_
: carbon dioxide
FI: forestomach indigestions
MB: methylene blue
MBRT: methylene blue reduction time
O_2_
: dioxygen
PCO_2_
: partial pressure in carbon dioxide
PVC: polyvinyl chloride
RJ: rumen juice
RJA: rumen juice analysis
*T*
: time
Temp: temperature

## 1 INTRODUCTION

Ruminants possess a unique digestive physiology, and healthy ruminal fermentation by the microbiota is of paramount importance to systemic animal health and productivity.^1^, ^2^ Forestomach indigestion (FI) can lead to gastrointestinal disturbances^3^ and life‐threatening diseases such as ruminal acidosis.^4^ Evaluating rumen juice (RJ) through a RJ analysis (RJA) is a simple tool for the identification of FI. Rumen juice analysis includes measuring the temperature, pH, methylene blue reduction time (MBRT), and assessing protozoal motility.^2^, ^5^, ^6^, ^7^ The MBRT measures the redox potential of the bacterial population of the rumen, which is predominantly Gram negative and anaerobic bacteria. The majority of the reactions occurring in the rumen are reductions,^8^ thus a prolonged MBRT indicates abnormally low microbial activity. Sampling techniques for RJ include rumenocentesis, oro/nasogastric intubation, specialized orogastric tubes,^9^ or via a rumenostomy site in cannulated animals.

The literature describing RJA is based on a minimum sample volume of 10 to 20 mL,^2^, ^3^, ^7^, ^10^, ^11^ however it might not be possible in all clinical cases to collect ≥10 mL of RJ without salivary contamination. Field practitioners might not have access to equipment to evaluate RJ within 10 minutes of collection, thus RJA can be delayed until samples reach basic laboratory equipment. Storing RJ at ambient temperature (15‐25°C or 60‐75°F) is optimal, but some of the RJA measurements change after 30 minutes.^12^ Additionally, there is a paucity of literature on the assessment of rumen protozoal activity. Current descriptions of adequate rumen protozoal activity are semiquantitative and described as: “5‐7 active protozoa/microscopic field at a ×100 magnification,”^11^ “≥40 protozoa per microscopic visual field,” and “>10 protozoa entering a single microscopic field over a period of 30 seconds at a ×40 magnification.”^7^, ^13^ These descriptions lack precision in both characterizing the activity of the protozoa as well as classifying their morphology. A novel scoring system has been proposed,^12^ and is based on the morphology of protozoa (large, medium, or small), their speed, trajectory, distance, and ciliary movements. Variations in RJA might lead to clinically important misinterpretation of FI. The effect of smaller samples (<10 mL) and delays between sampling and RJA on variables has not been reported, and the lack of a validated protozoal motility scoring system represents a limitation of RJA.

The objectives of this study were to quantify the effect of sample volume and lag time‐to‐analysis on RJA (temperature, pH, MBRT, and protozoal motility score), and determine the inter‐rater agreement of a novel protozoal motility scoring system.^12^ We hypothesized that smaller samples (≤5 mL) and samples with an increasing time to analysis (30 and 60 minutes) will have a higher pH, lower temperature, prolonged MBRT, and lower protozoal motility in comparison with larger samples (≥10 mL) and samples analyzed within 5 to 10 minutes of harvesting (0 minute).

## 2 MATERIALS AND METHODS

### 2.1 Study design

Nonrandomized prospective experimental ex vivo study. Rumen juice sampling and all steps of RJA were performed by the PI (S.A.C. Clergue) and 2 trained operators.

### 2.2 Animals

The 26 RJ samples were obtained from a single, 4‐year‐old, nonpregnant and nonlactating, rumen cannulated Holstein cow between April 2019 and January 2020. The cow was housed in a dry lot at the Veterinary Medical Teaching Hospital (VMTH) of University of California Davis and fed grass and alfalfa hay only with free access to water. Sampling for this study was approved by the UC Davis Institutional Animal Care and Use Committee (IACUC; #21078). The samples were harvested at 0900 and 1800 hours, consistently 4 hours after feeding (0500 and 1400 hours) to reach the nadir pH of RJ.^5^, ^9^ Rumen juice sampling consisted of inserting a fenestrated PVC pipe with a smooth closed bottom, through the rumen cannula in the dorsal sac, down to the ventral sac of the rumen and aspirating >2 L of rumen juice via flexible PVC tubing and 300 mL nylon dosing syringe.^9^ Aspirated RJ was collected into a 10L plastic bucket and transported to the laboratory on‐site for analysis within a few minutes.

### 2.3 Sampling and storage

**FIGURE 1:**
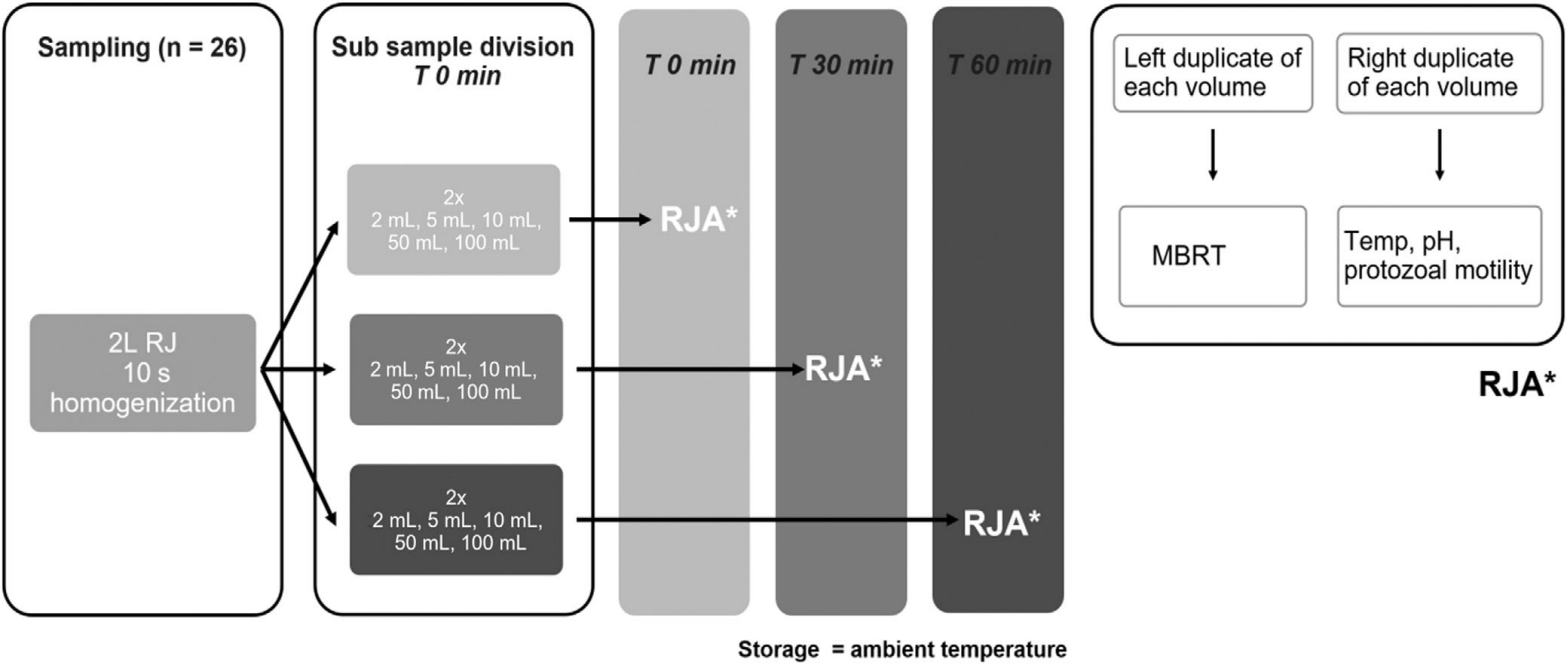
Diagram summarizing the subsampling, storage, and timing of analysis of 26 RJ samples obtained from a rumen cannulated cow, and an overview of the RJA steps as performed. MBRT, methylene blue reduction time; RJ, rumen juice; RJA, rumen juice analysis; *T*, time; Temp, temperature.

Each 2 L of RJ was divided into 3 sets of subsamples for analysis at 0, 30, and 60 minutes after sample collection (*T*_0_, *T*_30_, and *T*_60_, respectively). Each time point consisted of 2 duplicate sample volumes of 2, 5, 10, 50, and 100 mL (Figure 1). The 2, 5, and 10 mL samples were collected and stored in 14 mL polystyrene round‐bottom tubes with lids (Falcon, Corning, Reynosa, Tamaulipas, Mexico). The 50 and 100 mL samples were collected and stored in 150 mL plastic, nonsterile, standard specimen containers with lids (Gent‐L‐Kare, Medegen Medical Products‐Interplast Group, Gallaway, Tennessee). Laboratory analyses were performed at room temperature (mean 14.9°C). At *T*_0_, a complete RJA of all samples was performed. The same analyses were repeated 30 and 60 minutes later.

### 2.4 Temperature and pH measurement

All sample volumes were homogenized by gently inverting containers for 10 seconds. The duplicates of 2, 5, 10, 50, and 100 mL samples were placed against a white background. The pH and temperature were measured with an electronic pH meter (pHTestr30, Oakton—Environmental Express, Charleston, South Carolina). The pH meter was immersed in each sample for 10‐20 seconds, values recorded, and rinsed in deionized water between each sample. The relative alkaline and acidic pH categorizations were defined using a reference range of 6 to 7.2 for a forage‐based diet.^2^ The reference range used for rumen fluid temperature was 39°C to 40°C.^8^

### 2.5 Assessment of bacterial reduction

Sample duplicates were arranged in pairs. The left sample duplicate was used to assess the redox potential of RJ, also referred to as MBRT; the right sample of the duplicate was used as an untreated RJ color control. A 10‐minute video recording (SM‐T580, Samsung Electronics, Seoul, South Korea) of samples was initiated when the diluted 0.03% methylene blue (MB) was mixed with RJ. The MB was added to the left sample of each duplicate in proportionate quantity (5% of MB per sample); 0.1, 0.25, 0.5, 2.5, and 5 mL of 0.03% diluted MB in the 2, 5, 10, 50, and 100 mL RJ samples, respectively. The MB volumes were measured using 1 mL syringes for 0.1, 0.25, and 0.5 mL; 3 mL syringes for 2.5 mL; and 6 mL syringes for 5 mL. All samples were homogenized again (with or without MB) by gentle inversion for 10 seconds. The videos were reviewed by the corresponding author and the MBRT recorded in seconds. Samples were considered completely reduced when the sample returned to the control RJ color (MB is colored when oxidized, and transparent when reduced). Samples with a MBRT of ≤6 minutes were considered to have adequate bacterial activity.^2^, ^5^, ^10^, ^11^

### 2.6 Assessment of protozoal presence and motility

While the MBRT was running, a drop of RJ was collected from the duplicate RJ sample (no MB, color control) and placed on a microscope slide on a digital camera microscope (S30L Microscope, Omax Co, Gyeonggi‐Do, South Korea) connected to a computer (MacBookAir, Apple Inc, Cupertino, California). Samples were viewed on the 4× objective, and focus position was adjusted to the image on the computer's screen. Three 20‐second videos of each test sample were recorded using a video recording application (PhotoBooth, Apple Inc, Cupertino, California). Each video was viewed from a different position on the slide to ensure capture of an average motility score across 3 locations on each slide (left lower corner, center of the slide, and right upper corner). All videos were saved and labeled in code to maintain blinded review of motility.

### 2.7 Novel motility scoring

The protozoal motility videos were reviewed by 2 investigators independently (S.A.C. Clergue and S.M. Depenbrock). Each protozoal population (small, medium, and large size protozoa) was scored individually. Each score was based on the speed, trajectory, distance, and ciliary movements of the protozoa. The protozoa scores ranged from 1 to 5, where 1 represented at least 90% of observed protozoa with normal motility, and 5 represented ≤10% of observed protozoa with a normal motility (Table 1). Healthy protozoal motility was defined as a score of 1 to 3 for each population; scores of 4 and 5 were defined as nonhealthy. Two distinct scoring systems were used for each observation; 1 system for both large and medium size protozoa, and a different system for smaller protozoa, as previously described^12^ (Table 1 and Supplementary Document 1).

**TABLE 1:** Detailed novel scoring system for large/medium protozoa and small protozoa of rumen juice, grouped by speed/trajectory.

Protozoa size	Large and medium				Small		
% Motility	Active	Slow	Rotating	Immobile	Active	Residual	Immobile
Score 1	≥90				≥90		
Score 2	60‐90	≤30	≤10		60–90	≤30	≤10
Score 3	30‐60	≤40	≤30		30–60	≤40	≤30
Score 4	10‐30	≤30	≤50	≤10	10–30	≤20	≤70
Score 5	≤10			≥90	≤10		≥90

*Note*: The overall motility assessment was based on the speed, trajectory, distance, and ciliary movements of the protozoa. Percentages are a subjective assessment of the visible protozoa under the optic microscope at the 4× objective. Scores considered as “healthy/normal” motility are highlighted in gray.

### 2.8 Data analysis

A sample size calculation was performed using JMP Pro version 14.2 (SAS Institute, Cary, North Carolina), based on 2 objective tests of the RJA (pH and MBRT). To achieve a power of 80% and using the smallest SD from preliminary results (118 seconds for MBRT and 0.13 for pH), the sample size required to detect a significant difference between measurements for both MBRT and pH was 26. Normality of data was determined by Shapiro‐Wilk test. Mean ± SD was reported when data were normally distributed whereas median and range were reported when data were not normally distributed. Comparison between continuous variables, including pH, MBRT, temperature; and the ordinal variable, protozoal motility scores, at different time points (0, 30, and 60 minutes) and volumes (2, 5, 10, 50, and 100 mL) was performed using analysis of variance when data were normally distributed or Kruskall‐Wallis test when data were not normally distributed. Each variable × volume × time combination was compared twice: to the same variable × volume combination at other times, and to the same variable × time combination for other sample volumes. A commercial software was used for data analysis (GraphPad Prism, La Jolla, California). A *P* < .05 was considered significant. Post hoc analysis was performed using Dunn's test with adjusted *P* values for multiple comparisons. Inter‐rater absolute agreement was determined by comparing protozoal motility scores from both investigators and calculating the percentage of samples with a zero difference in score^14^ (referred to as absolute agreement). Absolute agreement was considered good if >75%.^14^ Due to the high likelihood of minor variation between motility scores between the 2 observers (because of the use of 2 scores per sample to account for difference sizes of protozoa), agreement was also calculated when there was a score difference of 0 or 1 (referred to as agreement).

## 3 RESULTS

Collection of RJ was performed on 26 days and no adverse effects were reported for the cow after collection. The pH, temperature, MBRT, and protozoal motility results were not normally distributed.

### 3.1 Temperature and pH

**FIGURE 2:**
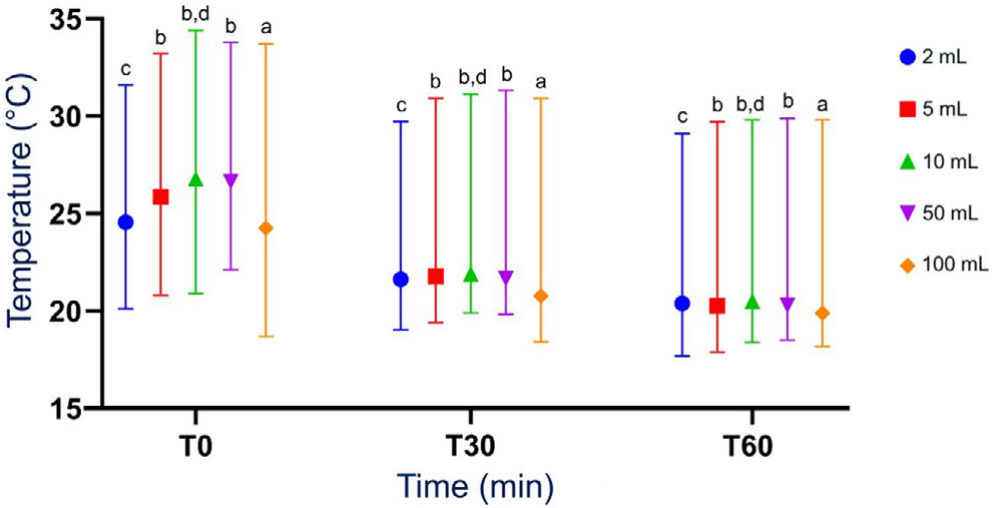
Median (range) temperature of all RJ sample volumes obtained from a rumen cannulated cow, measured as part of a RJA after 0 minute (*T*_0_), 30 minutes (*T*_30_), and 60 minutes (*T*_60_). N = 26. All median values with the same superscript letter are not significantly different from one another. The median temperature of 100 mL (a) samples was significantly lower (*P* = .01) than the median temperatures of the 5, 10, and 50 mL samples (b) at each time point (*T*_0_, *T*_30_, and *T*_60_; 24.3°C vs 25.9°C, 26.8°C, and 26.7°C respectively at *T*_0_; 20.8°C vs 21.8°C, 21.9°C, and 21.7°C respectively at *T*_30_; 19.9°C vs 20.3°C, 20.5°C, and 20.3°C respectively at *T*_60_). The median temperatures of 2 mL samples (c) were significantly lower (*P* = .01) than 10 mL samples (d) at each time point (*T*_0_, *T*_30_, and *T*_60_; 24.6°C vs 26.8°C at *T*_0_; 21.6°C vs 21.9°C at *T*_30_; 20.4°C vs 20.5°C at *T*_60_). RJ, rumen juice; RJA, rumen juice analysis; *T*, time.

All sample temperatures were below the reference range at all time points, and temperature decreased over time for all sample volumes (Figure 2). The median temperatures at *T*_0_ were significantly higher than the *T*_30_ (*P* = .01 for 2 mL, *P* < .001 for other volumes) and *T*_60_ median temperatures (*P* < .001). The *T*_30_ median temperatures were significantly higher (*P* = .01) than the *T*_60_ median temperatures, for all sample volumes (Figure 2). The median temperature of 100 mL samples was significantly lower (*P* = .01) than the median temperatures of the 5, 10, and 50 mL samples at all times (Figure 2 and Table S1). The median temperatures of 2 mL samples were significantly lower than 10 mL samples at all time points. Ten‐milliliter samples had the highest median temperatures at all time points (Figure 2 and Table S1).

**FIGURE 3:**
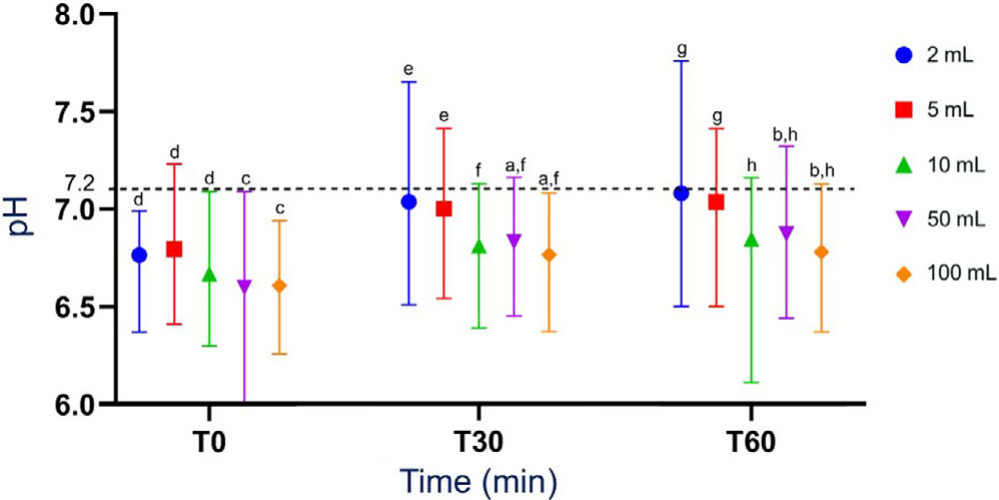
Median (range) pH of all RJ sample volumes obtained from a rumen cannulated cow, measured as part of a RJA after 0 minute (*T*_0_), 30 minutes (*T*_30_), and 60 minutes (*T*_60_). N = 26. Interrupted line: upper threshold of the normal pH range at 7.2 (lower threshold pH range is 6). All median values with the same superscript letter are not significantly different from one another. Median pH at *T*_60_ (b) was significantly higher than median pH at *T*_30_ (a) for 50 and 100 mL samples (*P* = .03 for 50 mL and *P* = .04 for 100 mL samples; 6.87 > 6.83 for 50 mL; and 6.78 > 6.76 for 100 mL). Median pH of the 2, 5, and 10 mL samples *(d)* were significantly higher than median pH of the 50 and 100 mL samples (c) at *T*_0_ (*P* = .01; 6.76, 6.79 and 6.67 > 6.60 and 6.61, respectively). Median pH of the 2 and 5 mL samples were significantly higher than pH of the 10, 50, and 100 mL samples at *T*_30_ and *T*_60_ (e > f [*P* = .01]; g > h [*P* = .01]; 7.03 and 7.0 > 6.81, 6.83, and 6.76 at *T*_30_; and 6.50 and 6.50 > 6.11, 6.44, and 6.37 at *T*_60_). RJ, rumen juice; RJA, rumen juice analysis; *T*, time.

**FIGURE 4:**
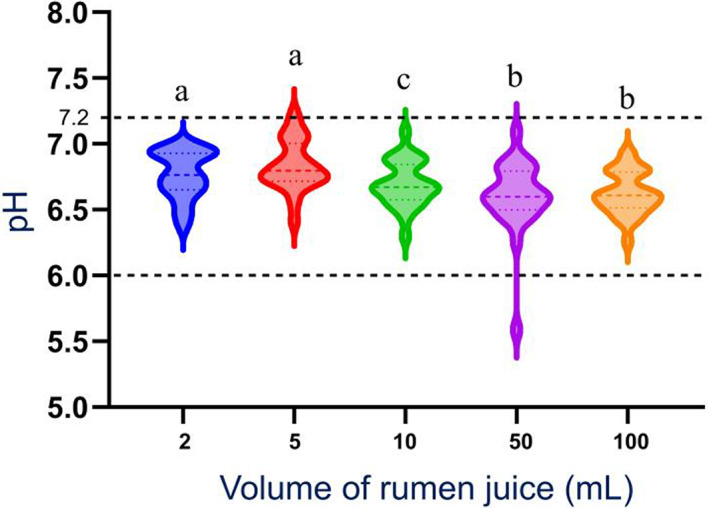
Violin plot of the distribution of the pH of all RJ sample obtained from a rumen cannulated cow, measured as part of a RJA after 0 minute (*T*_0_). N = 26 for each sample volume. Within each violin, the dashed line represents the median, and the upper and lower dotted lines represent the 75th and 25th percentiles, respectively. The dashed lines outside of the violin plots represent the upper and lower threshold of the reference interval for pH range of 6 and 7.2. Violin plots with different letter superscripts are different at *P* < .05. RJ, rumen juice; RJA, rumen juice analysis.

**FIGURE 5:**
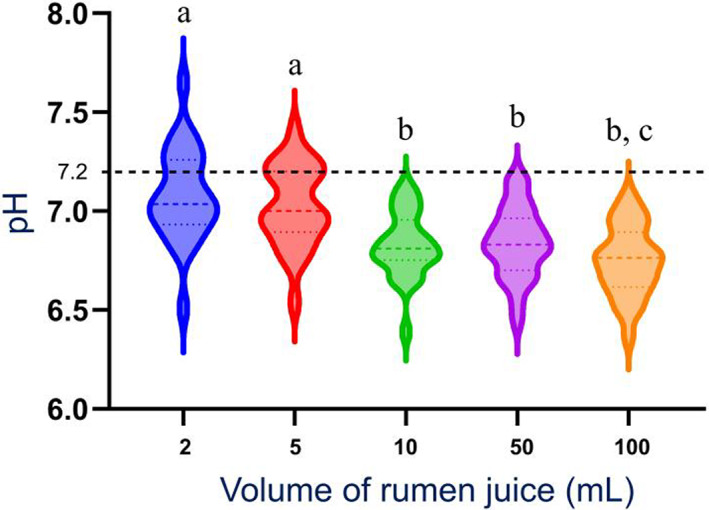
Violin plot of the distribution of the pH of all RJ sample volumes obtained from a rumen cannulated cow, measured as part of a RJA after 30 minutes (*T*_30_). N = 26 for each sample volume. Within each violin, the dashed line represents the median, and the upper and lower dotted lines represent the 75th and 25th percentiles, respectively. The dashed lines outside of the violin plots represent the upper and lower threshold of the reference interval for pH range of 6 and 7.2. Violin plots with different letter superscripts are different at *P* < .05. RJ, rumen juice; RJA, rumen juice analysis; *T*, time.

All median pH values were within the reference range (6.0‐7.2) at all time points. There was a significant increase (*P* < .05) in pH over time for each sample volume, with median pH at *T*_0_ always lower than median pH at *T*_30_ and *T*_60_ (Figure 3). For 50 and 100 mL samples only, median pH at *T*_60_ was significantly higher than median pH at *T*_30_ (Figure 3 and Table S2). At *T*_0_, pH of the 2, 5, and 10 mL samples were significantly higher than the pH of the 50 and 100 mL samples (Figures 3 and 4). At *T*_30_ and *T*_60_, pH of the 2 and 5 mL samples were significantly higher than pH of the 10, 50, and 100 mL samples (Figures 3 and 5). There was no significant difference between median pH of 2 and 5 mL samples, at each time point (Figures 4 and 5).

### 3.2 Assessment of bacterial reduction

**FIGURE 6:**
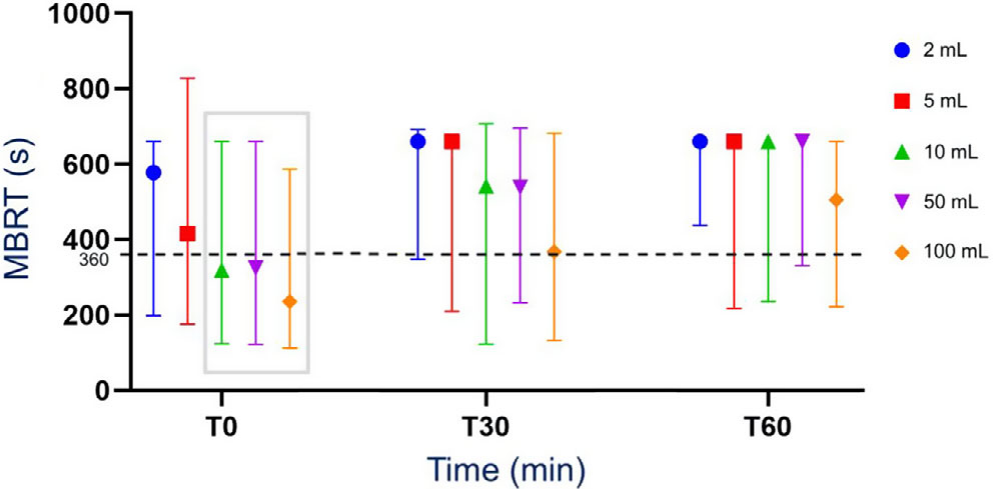
Median (range) MBRT of all RJ sample volumes obtained from a rumen cannulated cow, measured as part of a RJA after 0 minute (*T*_0_), 30 minutes (*T*_30_), and 60 minutes (*T*_60_). N = 26. Dotted line: upper threshold of the normal MBRT range at 360 seconds (6 minutes). Gray box outlines sample volumes and time where MBRT remained normal. MBRT, methylene blue reduction time; RJ, rumen juice; RJA, rumen juice analysis; *T*, time.

Median MBRT increased (*P* < .05) over time for all sample volumes; median MBRT at *T*_60_ were significantly longer (*P* = .01 for 2 mL, *P* < .01 for other volumes) than at *T*_0_ for all sample volumes (Figure 6). For 2, 5, and 50 mL samples, median MBRT at *T*_30_ were significantly longer (*P* = .01, *P* = .02, and *P* < .001, respectively) than at *T*_0_. For 10 mL samples, median MBRT at *T*_60_ was significantly longer than at *T*_30_ (*P* = .02). For 100 mL samples, median MBRT at *T*_60_ was significantly longer (*P* = .01) than at *T*_30_, and median MBRT at *T*_30_ was significantly longer (*P* = .01) than at *T*_0_.

**FIGURE 7:**
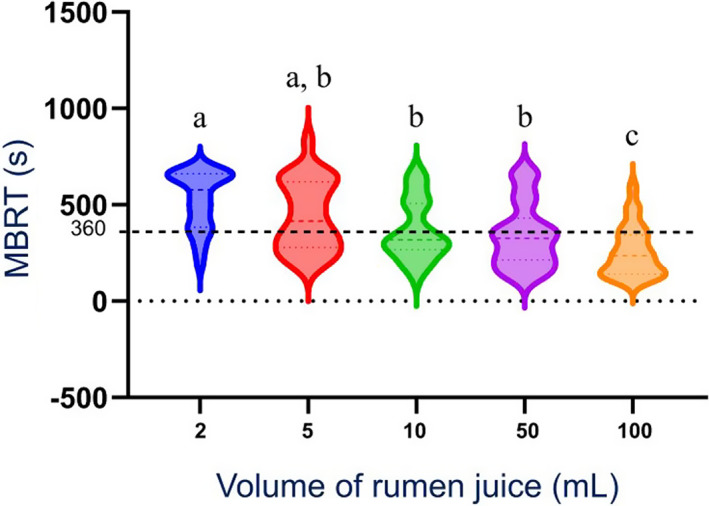
Violin plot of the distribution of the MBRT of all RJ sample volumes obtained from a rumen cannulated cow, measured as part of a RJA after 0 minute (*T*_0_). N = 26 for each sample volume. Dotted line: upper threshold of the normal MBRT range at 360 seconds (6 minutes). The interrupted line within each violin represents the median, and the dotted line the 25th percentile. Violin plots with different letter superscripts are different at *P* < .05. MBRT, methylene blue reduction time; RJ, rumen juice; RJA, rumen juice analysis; *T*, time.

Only the 10, 50, and 100 mL samples at *T*_0_ had a median MBRT within reference range (≤6 minutes). Median MBRT was significantly more rapid for 100 mL samples compared to all sample sizes (*P* < .001 for ≤5 mL samples and *P* = .01 for ≥10 mL samples) at *T*_0_ (Figure 7). Median MBRT was significantly more rapid for 100 mL samples compared to 2, 5, and 50 mL samples at *T*_30_ (*P* < .001, *P* < .001, and *P* = .01, respectively) and *T*_60_ (*P* < .001, *P* = .01, and *P* = .02, respectively). Median MBRT was significantly longer (*P* < .001) for 2 mL samples compared to large sample volumes (10, 50, and 100 mL) at *T*_0_ (Figure 7).

### 3.3 Assessment of protozoal presence and motility

Median protozoal scores for all protozoa size, volume samples, and times are summarized in Table S3.

### 3.4 Small and medium protozoa

There was no significant difference (*P* > .05) in median protozoal motility scores between sample volumes or times for medium and small protozoa, as determined by both investigators. For small protozoa, the median score was 4 at all collection time points and for all sample volumes, except for 100 mL sample at *T*_0_ for which 1 investigator (Suzanne A. C. Clergue) reported a median score of 4.5 (*P* = .03). For medium protozoa, the median score was 4 at all collection time points and for all sample volumes, for both investigators.

### 3.5 Large protozoa

The only significant difference noted by both investigators was that the motility score of large protozoa for ≤5 mL samples was significantly higher (lower protozoal motility) than 100 mL samples at *T*_60_ (*P* < .05). Investigator S.A.C. Clergue scored large protozoa from 2 mL samples significantly higher (*P* = .02; lower protozoal motility) than 50 mL samples at *T*_0_, and small sample volumes (2 and ≤5 mL respectively) significantly higher than ≥50 mL samples at *T*_30_ (*P* = .04 and *P* = .02 respectively) and *T*_60_ (*P* = .01 and *P* = .03 respectively). Investigator S.M. Depenbrock scored small sample volumes (2 and 5 mL) significantly higher (*P* = .01) than 100 mL samples at *T*_60_. The median score for large protozoa was 4 at all collection time points and for all sample volumes except for the 2 mL sample at *T*_60_, for which the median score was 5 for both investigators; and for the 5 mL sample at *T*_60_, for which the median score was 4.5 for 1 investigator (S.M. Depenbrock).

Inter‐rater absolute agreement (defined as >75% to <90% of samples with a 0 point score difference) between both investigators varied greatly between 30% and 88%. Overall, the absolute agreement was better for medium and large protozoa (consistently >50%) than for small protozoa. Good absolute agreement was achieved for the following categories: small protozoa of 100 mL samples at *T*_60_, medium protozoa of 2 mL samples at *T*_0_ and *T*_30_, medium protozoa of 10 mL and 100 mL samples at *T*_60_, large protozoa of 10 mL and 50 mL samples at *T*_0_, large protozoa of 5 mL samples at *T*_30_ (Table 2). The median score difference (excluding score differences of 0) was 1. Agreement defined as the percentage of samples with a 0 or 1 point score difference was >75% for all protozoa sizes, all sample volumes, and at all times.

**TABLE 2:** Absolute agreement between the 2 investigators of the novel rumen juice protozoal motility scoring system, calculated as the percentage of samples with a zero point difference in score.

Protozoa size	Large			Medium			Small		
Time (mL)	*T*_0_	*T*_30_	*T*_60_	*T*_0_	*T*_30_	*T*_60_	*T*_0_	*T*_30_	*T*_60_
2	52% (n = 25)	60% (n = 25)	69.6% (n = 23)	76.9% (n = 26)	76.9% (n = 26)	61.5% (n = 26)	66.7% (n = 24)	60% (n = 25)	60% (n = 25)
5	70.8% (n = 24)	78.3% (n = 23)	60% (n = 25)	61.5% (n = 26)	61.5% (n = 26)	61.5% (n = 26)	33.3% (n = 24)	60% (n = 25)	58.3% (n = 24)
10	76.9% (n = 26)	60% (n = 25)	61.5% (n = 26)	65.4% (n = 26)	57.7% (n = 26)	76.9% (n = 26)	56.6% (n = 23)	40% (n = 25)	70.8% (n = 24)
50	80.7% (n = 26)	61.5% (n = 26)	69.2% (n = 26)	61.5% (n = 26)	69.2% (n = 26)	68.2% (n = 26)	30.4% (n = 23)	52% (n = 25)	80% (n = 25)
100	53.8% (n = 26)	61.5% (n = 26)	61.5% (n = 26)	65.4% (n = 26)	65.4% (n = 26)	88.5% (n = 26)	37.5% (n = 24)	48% (n = 25)	36% (n = 25)

*Note*: Rumen juice samples were obtained from a rumen cannulated donor cow and scored as part of a RJ analysis after 0 minute (*T*_0_), 30 minutes (*T*_30_), and 60 minutes (*T*_60_). Number of samples scored per each category specified between parentheses. Gray areas represent the categories for which absolute agreement was acceptable (>75%). Data presented for all 3 protozoa type (large, medium, and small) and all sample volumes after 0 minute (*T*_0_), 30 minutes (*T*_30_), and 60 minutes (*T*_60_).

## 4 DISCUSSION

The results of this study demonstrate that smaller RJ samples (≤5 mL) and 30‐ to 60‐minute delays can adversely affect RJA results. Smaller samples (≤5 mL) had a higher pH in comparison to ≥50 mL samples at *T*_0,_ and in comparison to ≥10 mL samples at *T*_30_ and *T*_60_. Smaller samples (2 mL) had a lower bacterial reductive activity in comparison to larger samples (≥10 mL) at *T*_0_. For large protozoa, smaller samples (≤5 mL) had lower protozoal motility assessments in comparison to 100 mL samples at *T*_60_. The results of this study are consistent with the hypotheses that small volumes <10 mL and delays in analysis of 30 or 60 minutes could alter RJA. More specifically, longer time and smaller sample volume resulted in abnormal RJA measurements for pH, MBRT, and large protozoal motility (as hypothesized); temperature, medium, and small protozoal motility assessment did not follow the same trend. These findings indicate potentially clinically relevant changes in RJA that could potentially lead to misidentification of FI. The absolute agreement for the novel protozoal motility scoring system was very variable, but acceptable for analysis of large‐ and medium‐sized protozoa.

The median temperature results were within the reference range of 39°C to 40°C.^8^ Although temperature assessment is not part of the clinical decision after RJA to manage FI, it is a marker of the environmental stress on the sample. The low ambient temperature (around 15°C) recorded in our study increased the likelihood for cold shock to flora and fauna in the RJ samples in comparison to the optimal temperature range (15°C‐25°C) reported.^12^ The median temperature of 10 mL samples was the highest at all time points, and the temperature significantly decreased for 2 mL in comparison to 10 mL samples only. This finding was unexpected, as temperature change was hypothesized to be proportionate to sample volume. This could be because of the change of sample container for which ≤10 mL samples were stored and analyzed in a 10‐mL Falcon tube, with minimal direct contact between the RJ and the ambient air and desk (1.76 cm^2^ approximately), because the tube is narrow. In contrast, ≥50 mL samples were stored and analyzed in 150 mL containers, with a larger contact surface of RJ to ambient air and laboratory surfaces (approximately 19.6 cm^2^). The 10 mL samples had the highest volume‐to‐contact surface ratio (5.7:1), followed by 100 mL samples (5.1:1), 5 mL samples (2.84:1), 50 mL samples (2.6:1), and 2 mL samples (1.1:1). However, this hypothesis does not explain why the median 100 mL samples' temperature was lower than other sample volumes. The reason for this finding remains unknown, especially as the pH meter (recording temperature) was immersed into the sample completely rather than measuring the surface temperature.

pH is the least subjective measurement in RJA, and the significant variations in pH observed in this study might have clinical relevance, despite all median pH results remaining within a reference range for cattle on a forage diet, for all sample volumes, and at all times. The variations observed in this study were on a healthy animal, however changes in pH outside the reference interval in a sick ruminant could be more critical to clinical decision making.

The majority of MBRT results were in accordance with the study hypothesis. It is important to note that at *T*_30_, the median MBRT of the 100 mL sample was only 6 seconds over the threshold of 6 minutes, indicating that 100 mL samples have a relatively small magnitude deficiency in reductive activity at 30 minutes. Under clinical test conditions, many 100 mL samples (or potentially larger samples) might still be interpreted correctly after 30 minutes.

The changes in temperature and air contact area could also have affected the pH and MBRT results because, as detailed by the Nernst equation, *E* (the reduction potential of an oxidation and reduction reaction) is a factor of the temperature and other factors, including the electrode potential, gas pressure, and concentration of the chemical species undergoing reduction and oxidation (including protons).^15^ Similarly, a larger contact surface between the sample and ambient air could lead to higher amounts of CO_2_ escaping the sample and more O_2_ oxidizing the sample, leading to alterations in pH (changes in PCO_2_) and MBRT (the lack of O_2_ in the rumen being key to having proper anaerobic bacterial activity); all study samples were analyzed at room temperature and in open containers.^13^ One, or a combination, of these phenomena could explain why the median pH of 10 mL at *T*_30_ and *T*_60_ were significantly lower than the median pH of 50 mL samples, and why at *T*_30_ and *T*_60_, all sample volumes (2, 5, and 50 mL) except 10 mL samples had a significantly higher MBRT than 100 mL samples.

Eighty‐six of 90 combinations of protozoa types, sample volume, time delay, and investigator had a similar median protozoal motility score of 4 (Table S3). The lack of difference between groups could be because of a flaw in the novel scoring system, although the same scoring system detected wider variations in another study,^12^ or a flaw in sample processing before motility assessment such as cold shock. It has been recommended that protozoal activity be assessed on a warm microscopic slide at 30°C,^13^ which was not performed in this study. Previous studies suggested that protozoa in RJ do not survive at temperatures below 26°C,^16^ which is warmer than the 15°C room temperature of our study setting. A potential alternative could be to have run the Lugol's iodine coloration in parallel to assess the starch granules of the protozoa as an estimation of the energy levels of the fauna.^13^ The RJ was not strained or squeezed at any point during or after collection, to limit the decline in protozoa count and activity.^17^ However, the high quantity of fiber particles could have limited protozoal movement, leading to an abnormally high median motility score, limited visibility, or a combination of the 2, especially of smaller protozoa. The limited visibility because of fiber particles made it impossible to score some of the protozoal populations of a few samples. Therefore, the number of sample volume × time data points varies between 23 and 26 for some of the protozoa motility scores (Table 2 and Table S3). Absolute agreement between the 2 investigators was suboptimal for 37 of 45 samples reviewed (combinations of protozoa types, sample volume, and time delay). This could have been secondary to a flaw in sample processing, or a flaw in the scoring system, making scores too difficult to distinguish from each other. Absolute agreement >75% was reached for some medium and large protozoa sample volumes. Absolute agreement for small protozoa was not reached, which is likely because of small protozoa being more difficult to identify and trace, leading to inconsistent scoring. When agreement was defined as the number of samples with a 0 or 1 point difference in score, agreement was >75% for all sample volumes, for all protozoa sizes, and time points. A score difference of 1 should have minimal clinical impact on the diagnosis of FI, as the protozoal motility is defined as healthy for scores going from 1 to 3 (Supplementary Document 1). If the 1 point score difference is between 3 and 4, that changes the assessment of the fauna viability. However, it is unlikely that a change in protozoa viability from score 3 to 4 alone could lead to misinterpreting a FI diagnosis, as the remainder of the RJA would be used for an assessment. Protozoa are the most sensitive to environmental changes,^2^, ^6^ and it is unlikely that a sample, even if “misclassified” as having a viable fauna with an overall score of 3 (instead of nonviable with an overall score of 4), would have a normal MBRT and pH. Alternatively, the scoring system could be simplified in future investigations to limit single point differences between observers.

One of the limitations of this study was the use of an experimental, unvalidated subjective motility scoring system based on qualitative (speed, trajectory, distance, and ciliary movements) rather than quantitative criteria for protozoal motility assessment. Future research could benefit from using software to automate quantification of motility into numeric values.^18^ However, using a visual score that only requires an optical microscope is more practically applicable in livestock clinic settings. Other novel techniques like real‐time PCR could be useful to assess the rumen protozoal biomass, but not to assess their viability.^19^ Another limitation was that the RJ was harvested from a cannulated cow. Other options commonly used in clinical settings to harvest RJ include using a naso or orogastric tube, and rumenocentesis.^9^, ^20^ However, intubation results in inconsistent sample collection sites within the rumen and salivary contamination.^2^, ^5^, ^6^ Rumenocentesis is the most accurate RJ sampling technique for pH measurement because of minimal salivary contamination and consistent anatomic placement in a ruminant,^2^, ^5^, ^6^, ^20^ but needles occasionally clog and thus insufficient sample volumes might be collected; repeated rumenocentesis can predispose the animal to peritonitis.^21^

The MBRT video recordings were reviewed by a single nonblinded investigator, and time of the experiment (*T*_0_, *T*_30_, and *T*_60_) could have biased the investigator. However, this bias was minimized by the quantifiable nature of the results (color change of the sample with exact time recording). There is a relative lack of research on RJA as a diagnostic tool for health status in the individual ruminant, as the majority of studies focus on how to maximize the feed intake and ruminal transformation of feed into energy,^22^, ^23^ or therapeutic aspects of transfaunation.^24^, ^25^, ^26^, ^27^ Although there are several studies describing RJA,^5^, ^6^, ^9^, ^10^, ^11^, ^13^, ^17^, ^25^ a validated RJA procedure is lacking. Subjective variables, such as color, odor, and viscosity of the RJ, have been described. Although these variables were observed and reported in the experimental phase of this study, there were no variations for all 26 samplings (color was olive green, odor was aromatic, and viscosity was appropriate for 100% of samples). Other variables have been described for RJA, including sedimentation testing, Gram coloration, protozoal number estimation, Lugol's iodine coloration for protozoa, chloride, lactate, and volatile fatty acid concentrations. These additional analyses were not performed in our RJA methodology. Our study selected a simple, practical subset of tests to ensure that all measurements were performed within minutes of their reference time (*T*_0_, *T*_30_, and *T*_60_) and would easily be repeatable in all clinical settings.

The clinical relevance of this study is that interpreting RJA from a <10mL sample, or waiting ≥30 minutes after sampling could yield falsely elevated pH, falsely low reductive activity of the bacteria, and falsely low motility of the protozoa. These changes could lead to an incorrect clinical diagnosis of FI. To preserve the maximum diagnostic accuracy of RJA, we recommend collecting a sample volume ≥10 mL, performing the RJA within 30 minutes and performing MBRT analysis first, because MBRT was relatively more sensitive to time. Additionally, a narrow contact surface between the ambient air and RJ, and minimal contact of the RJ container to surfaces (like a table) are likely important when measuring accurate temperature, pH, and MBRT of RJ, as well as to limit cold shock to rumen microbes in the sample. The novel motility scoring system described might be useful for medium and large protozoa, and requires validation by comparing it to another objective quantitative technique.

## CONFLICT OF INTEREST DECLARATION

Munashe Chigerwe serves as Associate Editor for the Journal of Veterinary Internal Medicine. He was not involved in review of this manuscript. No other authors declare a conflict of interest.

Authors declare no conflict of interest.

## OFF‐LABEL ANTIMICROBIAL DECLARATION

Authors declare no off‐label use of antimicrobials.

## INSTITUTIONAL ANIMAL CARE AND USE COMMITTEE (IACUC) OR OTHER APPROVAL DECLARATION

Approved by the IACUC of University of California Davis (#21078).

## HUMAN ETHICS APPROVAL DECLARATION

Authors declare human ethics approval was not needed for this study.

## Supporting information


**Data S1:** Supporting Information.

**Table S1:** Median temperature in °C of all RJ sample volumes obtained from a rumen cannulated cow, measured as part of a RJA after 0 minute (*T*_0_), 30 minutes (*T*_30_), and 60 minutes (*T*_60_). N = 26. All median values with the same superscript letter are not significantly different from one another. The median temperature of 100 mL (a) samples was significantly lower (*P* = .01) than the median temperatures of the 5, 10, and 50 mL samples (b) at each time point (*T*_0_, *T*_30_, *T*_60_). The median temperatures of 2 mL samples (c) were significantly lower (*P* = .01) than 10 mL samples (d) at each time point (*T*_0_, *T*_30_, *T*_60_). RJ, rumen juice; RJA, rumen juice analysis; *T*, time.

**Table S2:** Median pH of all RJ sample volumes obtained from a rumen cannulated cow, measured as part of a RJA after 0 minute (*T*_0_), 30 minutes (*T*_30_), and 60 minutes (*T*_60_). N = 26.

**Table S3:** Median RJ protozoal motility score for each investigator (SC and SD). RJ samples were obtained from a rumen cannulated donor cow and scored as part of a RJA after 0 minute (*T*_0_), 30 minutes (*T*_30_), and 60 minutes (*T*_60_). Number of samples scored per each category specified between parenthesis (n = x). Data presented for all 3 protozoa type (large, medium, and small) and all sample volumes after 0 minute (*T*_0_), 30 minutes (*T*_30_), and 60 minutes (*T*_60_). RJ, rumen juice; RJA, rumen juice analysis; *T*, time.
