# Supplementary material for: Effect of sample volume and time on rumen juice analysis in cattle
Source: J Vet Intern Med. 2023 Apr 7;37(3):1262–70. doi: 10.1111/jvim.16697 (PMC10229352; doi:10.1111/jvim.16697)
Supplement: Supplementary file 1 — Data S1: Supporting Information. [file JVIM-37-1262-s004.pdf]

LARGE AND MEDIUM SIZE PROTOZOA

1) - ≥ 90 % protozoa: long distance on the slide, high relative speed and linear movements (few direction changes).
2) - 60-90 % protozoa: linear high speed movements, long distances

   - ≤ 30 % protozoa: cross slowly the slide with curvilinear movements

   - ≤ 10 % protozoa: rotating on themselves or in a small portion of the slide, ciliary motility still visible
3) - 30-60% protozoa: linear high speed movements, long distances

   - ≤ 40% protozoa: cross slowly the slide with curvilinear movements

   - ≤ 30% protozoa: rotating on themselves or in a small portion of the slide, ciliary motility still visible
4) - ≤ 50 % protozoa: rotating on themselves or in a small portion of the slide, ciliary motility still visible

   - ≤ 30 % protozoa: cross slowly the slide with curvilinear movements

   - 10-30% protozoa: linear high speed movements, long distances

   - ≤ 10% protozoa: completely motionless (cilia comprised)
5) - ≥ 90% protozoa: completely motionless (cilia comprised)

   - ≤ 10% protozoa: residual motility of any sort

SMALL SIZE PROTOZOA

1) - ≥ 90% protozoa: small distances on the slide, very high relative speed and numerous direction changes.

2) - 60-90 % protozoa: small distances, high speed, numerous direction changes

- ≤ 30% protozoa: slow residual movements in a very restricted portion of the slide, ciliary motility still visible

- ≤ 10% protozoa: completely motionless (cilia comprised)

3) - 30-60% protozoa: small distances, high speed, numerous direction changes

- ≤ 40% protozoa: slow residual movements in a very restricted portion of the slide, ciliary motility still visible

- ≤ 30% protozoa: completely motionless (cilia comprised)

4) - ≤ 70 % protozoa: completely motionless (cilia comprised)

- ≤ 20 % protozoa: slow residual movements in a very restricted portion of the slide, ciliary motility still visible

- 10-30 % protozoa: small distances, high speed, numerous direction changes

5) - ≥ 90% protozoa: completely motionless (cilia comprised)

- ≤ 10% protozoa: residual motility of any sort

Supplementary Document 1: Detailed novel scoring system for system for large/medium protozoa and small protozoa of rumen juice, with specific assessments of the speed, trajectory, distance and ciliary movements of the protozoa. Percentages are a subjective assessment of the visible protozoa under the optic microscope at the 4× objective.
