# Supplementary material for: Effect of sample volume and time on rumen juice analysis in cattle
Source: J Vet Intern Med. 2023 Apr 7;37(3):1262–70. doi: 10.1111/jvim.16697 (PMC10229352; doi:10.1111/jvim.16697)
Supplement: Supplementary file 2 — Table S1: Median temperature in °C of all RJ sample volumes obtained from a rumen cannulated cow, measured as part of a RJA after 0 minute (T 0), 30 minutes (T 30), and 60 minutes (T 60). N = 26. All median values with the same superscript letter are not significantly different from one another. The median temperature of 100 mL (a) samples was significantly lower (P = .01) than the median temperatures of the 5, 10, and 50 mL samples (b) at each time point (T 0, T 30, T 60). The median temperatures of 2 mL samples (c) were significantly lower (P = .01) than 10 mL samples (d) at each time point (T 0, T 30, T 60). RJ, rumen juice; RJA, rumen juice analysis; T, time. [file JVIM-37-1262-s003.pdf]

Sample volume	Temperature T_0	Temperature T_{30}	Temperature T_{60}
**2 mL**	24.6 [c]	21.6 [c]	20.4 [c]
**5 mL**	25.9 [b]	21.8 [b]	20.3 [b]
**10mL**	26.8 [b, d]	21.9 [b, d]	20.5 [b, d]
**50 mL**	26.7 [b]	21.7 [b]	20.3 [b]
**100 mL**	24.3 [a]	20.8 [a]	19.9 [a]

Supplementary Table 1: Median temperature in °C of all RJ sample volumes obtained from a rumen cannulated cow, measured as part of a RJA after 0min (T_0), 30min (T_{30}) and 60min (T_{60}). N = 26. All median values with the same superscript letter are not significantly different from one another. The median temperature of 100mL *(a)* samples was significantly lower (*P* = 0.01) than the median temperatures of the 5, 10 and 50mL samples *(b)* at each time point (T_0, T_{30},T_{60}). The median temperatures of 2mL samples *(c)* were significantly lower (*P* = 0.01) than 10mL samples (d) at each time point (T_0, T_{30},T_{60}). RJ (rumen juice), RJA (rumen juice analysis), T (time), mL (milliliters).
