# Supplementary material for: Effect of sample volume and time on rumen juice analysis in cattle
Source: J Vet Intern Med. 2023 Apr 7;37(3):1262–70. doi: 10.1111/jvim.16697 (PMC10229352; doi:10.1111/jvim.16697)
Supplement: Supplementary file 3 — Table S2: Median pH of all RJ sample volumes obtained from a rumen cannulated cow, measured as part of a RJA after 0 minute (T 0), 30 minutes (T 30), and 60 minutes (T 60). N = 26. [file JVIM-37-1262-s001.pdf]

**Sample volume**	**pH T_0**	**pH T_{30}**	**pH T_{60}**
**2 mL**	6.77 [d]	7.04 [e]	7.08 [g]
**5 mL**	6.80 [d]	7.00 [e]	7.04 [g]
**10mL**	6.67 [d]	6.81 [f]	6.85 [h]
**50 mL**	6.60 [c]	6.83 [a, f]	6.87 [b, h]
**100 mL**	6.61 [c]	6.77 [a, f]	6.78 [b, h]

Supplementary Table 2: Median pH of all RJ sample volumes obtained from a rumen cannulated cow, measured as part of a RJA after 0min (T_0), 30min (T_{30}) and 60min (T_{60}). N=26.

All median values with the same superscript letter are not significantly different from one another. Median pH at T_{60} *(b)* was significantly higher than median pH at T_{30} *(a)* for 50 and 100mL samples (P = 0.03 for 50mL and = 0.04 for 100mL samples). Median pH of the 2mL, 5mL and 10mL samples *(d)* were significantly higher than median pH of the 50mL and 100mL samples *(c)* at T_0 ($P = 0.01$). Median pH of the 2mL and 5mL samples were significantly higher than pH of the 10mL, 50mL and 100mL samples at T_{30} and T_{60} (e > f ($P = 0.01$); g > h ($P = 0.01$)). RJ (rumen juice), RJA (rumen juice analysis), T (time), mL (milliliters).
