# Supplementary material for: Effect of sample volume and time on rumen juice analysis in cattle
Source: J Vet Intern Med. 2023 Apr 7;37(3):1262–70. doi: 10.1111/jvim.16697 (PMC10229352; doi:10.1111/jvim.16697)
Supplement: Supplementary file 4 — Table S3: Median RJ protozoal motility score for each investigator (SC and SD). RJ samples were obtained from a rumen cannulated donor cow and scored as part of a RJA after 0 minute (T 0), 30 minutes (T 30), and 60 minutes (T 60). Number of samples scored per each category specified between parenthesis (n = x). Data presented for all 3 protozoa type (large, medium, and small) and all sample volumes after 0 minute (T 0), 30 minutes (T 30), and 60 minutes (T 60). RJ, rumen juice; RJA, rumen juice analysis; T, time. [file JVIM-37-1262-s002.pdf]

Protozoa size	Large		Medium		Small	
**Investigator**	SC	SD	SC	SD	SC	SD
**2mL - T_0**	4 (n=26)	4 (n=25)	4 (n=26)	4 (n=26)	4 (n=24)	4 (n=26)
**2mL - T_{30}**	4 (n=25)	4 (n=26)	4 (n=26)	4 (n=26)	4 (n=25)	4 (n=26)
**2mL - T_{60}**	5 (n=24)	5 (n=24)	4 (n=26)	4 (n=26)	4 (n=25)	4 (n=26)
**5mL - T_0**	4 (n=24)	4 (n=26)	4 (n=26)	4 (n=26)	4 (n=24)	4 (n=26)
**5mL - T_{30}**	4 (n=23)	4 (n=26)	4 (n=26)	4 (n=26)	4 (n=25)	4 (n=25)
**5mL - T_{60}**	4 (n=25)	4.5 (n=26)	4 (n=26)	4 (n=26)	4 (n=24)	4 (n=26)
**10mL - T_0**	4 (n=26)	4 (n=26)	4 (n=26)	4 (n=26)	4 (n=24)	4 (n=25)
**10mL - T_{30}**	4 (n=25)	4 (n=26)	4 (n=26)	4 (n=26)	4 (n=25)	4 (n=26)
**10mL - T_{60}**	4 (n=26)	4 (n=26)	4 (n=26)	4 (n=26)	4 (n=24)	4 (n=26)
**50mL - T_0**	4 (n=26)	4 (n=26)	4 (n=26)	4 (n=26)	4 (n=23)	4 (n=25)
**50mL - T_{30}**	4 (n=26)	4 (n=26)	4 (n=26)	4 (n=26)	4 (n=25)	4 (n=26)
**50mL - T_{60}**	4 (n=26)	4 (n=26)	4 (n=26)	4 (n=26)	4 (n=26)	4 (n=25)
**100mL - T_0**	4 (n=26)	4 (n=26)	4 (n=26)	4 (n=26)	4.5 (n=26)	4 (n=24)
**100mL - T_{30}**	4 (n=26)	4 (n=26)	4 (n=26)	4 (n=26)	4 (n=25)	4 (n=26)
**100mL - T_{60}**	4 (n=26)	4 (n=26)	4 (n=26)	4 (n=26)	4 (n=25)	4 (n=26)

Supplementary Table 3: Median RJ protozoal motility score for each investigator (SC and SD). RJ samples were obtained from a rumen cannulated donor cow and scored as part of a RJA after 0min (T_0), 30min (T_{30}) and 60min (T_{60}). Number of samples scored per each category specified between parenthesis (n=x). Data presented for all three protozoa type (large, medium and small) and all sample volumes after 0min (T_0), 30min (T_{30}) and 60min (T_{60}). RJ (rumen juice), RJA (rumen juice analysis), T (time), mL (milliliters).
